# Predictive Modeling of Juvenile Smalltooth Sawfish Habitats: Challenges and Opportunities for Conservation

**DOI:** 10.1002/ece3.70592

**Published:** 2025-01-21

**Authors:** Andrea M. Kroetz, Simon Dedman, John K. Carlson

**Affiliations:** ^1^ Cooperative Institute for Marine and Atmospheric Studies, Rosenstiel School for Marine and Atmospheric Science University of Miami Miami Florida USA; ^2^ National Marine Fisheries Service Southeast Fisheries Science Center Panama City Florida USA; ^3^ Department of Biological Sciences, Institute of Environment Florida International University Miami Florida USA

**Keywords:** conservation, elasmobranch, endangered species, habitat use, predictive spatial modeling, sawfish, species distribution modeling

## Abstract

Effective conservation of rare species necessitates the identification of critical habitats and their specific features that influence species occurrence. This study focused on smalltooth sawfish ( *Pristis pectinata* ), a critically endangered elasmobranch, to explore how predictive spatial modeling can enhance conservation efforts. By leveraging long‐term occurrence and relative abundance data from scientific gillnet surveys, along with in situ environmental data, we used boosted regression trees (BRT) to pinpoint key habitat features essential for juvenile sawfish. Our analysis revealed strong correlations between sawfish presence and environmental variables, with a preferential selection of very shallow, warm, and saline waters fringed with mangroves, particularly those with high pneumatophore density. High relative abundances were observed in warmer months, and predictions of presence were consistent around discrete mangrove‐lined areas in Everglades National Park throughout all seasons. This study emphasizes the importance of high‐quality environmental data in predictive modeling and informs management strategies aimed at protecting the critical habitats necessary for the recovery of this species. Preventing the loss of mangroves in vulnerable regions of the smalltooth sawfish's range—especially near anthropogenic influences such as the Charlotte Harbor Estuary—is crucial for recovery. We also highlight the need for improved data access to facilitate global abundance predictions, thereby enhancing spatial management and conservation efforts for rare species.

## Graphical abstract

This study identifies key habitat features essential for the conservation of endangered juvenile smalltooth sawfish through predictive spatial modeling, revealing that juvenile sawfish prefer very shallow, warm, and saline waters adjacent to mangroves with dense pneumatophores. The findings underscore the importance of protecting these habitats from human impact to support the species' recovery. Additionally, the research emphasizes the importance of high‐quality environmental data in developing effective conservation strategies.

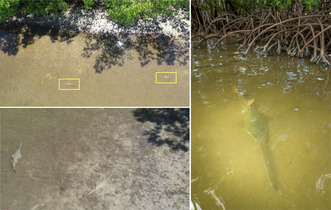

## 1 Introduction

Effective conservation of a species relies in part on the identification of essential habitats and specific features of habitats that may influence occurrence. Comprehensive knowledge of species distributions and habitat use is essential for conservation planning, species recovery, and informing management objectives (Norse and Crowder 2005; Sobel and Dahlgren 2004; Elith and Leathwick 2009; Guisan et al. 2013). For decades, marine ecosystems have been under global pressure from anthropogenic disturbances such as climate change and habitat modification/degradation (Lotze et al. 2006; Pauly and Zeller 2016), leading to conservation concerns for many species globally. For rare species (e.g., threatened or endangered species) and species whose historic range has been significantly reduced, identifying specific habitat features that may be crucial to their conservation and recovery are of vital importance. This is especially true for vulnerable large‐bodied, coastal‐associated species such as elasmobranchs.

Predictive spatial modeling can identify distributions and important habitats for species, making these models invaluable for conservation and spatial ecology (Guisan and Thuiller 2005; Elith and Leathwick 2009). Environmental variables influencing species distributions, and species response to these variables, are necessary components of conservation planning (Block and Brennan 1993; Sobel and Dahlgren 2004; MacKenzie et al. 2006) and need to be considered when designing and implementing management actions and recovery plans for rare species. However, these data are limited or absent for many rare marine species, creating conservation challenges (Newbold 2010; Marcer et al. 2013). Inherently, rare species are difficult to locate and are often data‐limited, creating limitations on resources to study the species and challenges in species distribution modeling (Elith et al. 2006; Guisan et al. 2006; Jeliazkov et al. 2022). For monitoring and conservation, rare species would benefit the most from predictive distribution modeling, yet the limited data available makes them the most difficult to model (Lomba et al. 2010) and ultimately difficult to enact conservation measures such as critical habitat designations and protections. Improving predictions of rare species distributions remains a challenge, and few models exist for marine species (Dedman et al. 2015; Aubry, Raley, and McKelvey 2017; Galante et al. 2018; Helmstetter et al. 2021).

Advances in statistical and spatial modeling have led to an increase in the use of model‐based approaches to identify essential and/or critical habitat for many species ranging from Ord's kangaroo rat ( *Dipodomys ordii* ), to threatened bird species such as Montagu's harrier ( *Circus pygargus* ), and to endangered elasmobranchs such as the Brazilian guitarfish ( *Rhinobatos horkelii* ) (Brotons, Manosa, and Estrada 2004; Fehérvári et al. 2012; Heinrichs et al. 2010; Klippel, Amaral, and Vinhas 2016). Model‐based approaches to identifying Essential Fish Habitat (i.e., waters and substrate necessary for fish spawning, breeding, feeding, or growth to maturity) have been used in fishery management applications (North Pacific Fishery Management Council 2016). Species distribution models (SDMs) predict environmental suitability for species in space and time and are frequently used to spatially predict environmental suitability (Elith and Leathwick 2009; Franklin 2010; Guisan and Thuiller 2005). SDMs are advantageous in data‐poor applications and can improve conservation efforts (Guisan et al. 2013) for data‐limited rare species. Boosted regression trees (BRTs) are a robust statistical tool and have increasingly been applied to ecological, marine, and rare marine case studies (Leathwick et al. 2006; Elith, Leathwick, and Hastie 2008; Froeschke, Stunz, and Wildhaber 2010; Dedman et al. 2015). As a model‐averaging ensemble method that allows for explanation and prediction (Elith, Leathwick, and Hastie 2008), BRTs are robust in that the model can accommodate continuous and categorical predictors, missing predictor values, and is unaffected by outliers and transformations. Correlations among explanatory variables are not an issue, as the model effectively parses out the influences and relationships of each variable with the response variable. Accommodating complex multivariate relationships, predictor responses can be modeled as interactions and inherently incorporated when necessary to improve the model fit to the cross‐validated data (Elith, Leathwick, and Hastie 2008). BRTs provide best‐in‐class performance and can be useful to both predict and understand the relationships between the predictors and the response variable (Elith et al. 2006; Valavi et al. 2022).

Among marine fishes, sawfishes (Pristidae) are among the most threatened with extinction; globally all five species are listed as critically endangered under the International Union for Conservation of Nature Red List of Threatened Species (IUCN; Harry et al. 2024). Once widely distributed throughout the Atlantic Ocean from the west coast of Africa and throughout the mid‐Atlantic states of the United States (U.S.) to Uruguay, smalltooth sawfish ( *Pristis pectinata* ) populations have dramatically declined, attributed to bycatch mortality, habitat loss, and the species' limited reproductive potential to offset losses (Brame et al. 2019; Carlson et al. 2022). However, potentially viable populations for the species now only exist in the southeastern U.S. (Brame et al. 2019), Mexico (Bonfil et al. 2018), the Bahamas (Guttridge et al. 2015), and Cuba (Figueredo Martín et al. 2013). In the U.S., initial research identified shallow, euryhaline nearshore habitats associated with red mangroves ( *Rhizophora mangle* ) to be essential to the conservation and recovery of the species as these habitats serve as nurseries (NMFS 2009; FR 45353; Norton et al. 2012; Wiley and Simpfendorfer 2010). Further refinement has identified juvenile smalltooth sawfish to have an affinity for shallow (< 1 m), warm (25°C–30°C) water with wide‐ranging salinities (18–39) (Poulakis et al. 2011; Simpfendorfer et al. 2011; Kroetz, Carlson, and Grubbs 2017). Though research has shown a correlation between sawfish and these habitat features, it is still largely unknown which specific habitat features and environmental factors are critical to the conservation and recovery of this species over smaller spatial scales and across different life stages.

To guide future research initiatives and conservation efforts for rare species, we tested our hypothesis that presence/absence data from scientific surveys, combined with in situ environmental data, can be modeled to identify specific habitat features that predict occurrences of rare species over a broad spatial area. We used smalltooth sawfish as our candidate species for this investigation, resulting in two main objectives for the study. First, we used a boosted regression tree model to analyze the relationship between juvenile smalltooth sawfish occurrence and relative abundance data from a long‐term scientific gillnet survey and the explanatory variables of concomitantly recorded in situ environmental data. This analysis revealed the most influential variables affecting the presence and abundance of smalltooth sawfish. Second, we aimed to obtain the same explanatory environmental variables across a wider spatial and temporal range in south Florida, enabling the model to predict key habitat features throughout different seasons. The results of this model can assist managers' refinement of the critical habitat features necessary for the conservation and recovery of this species. Habitat loss from both anthropogenic destruction and climate change poses a threat to smalltooth sawfish across their range, and our findings have broad applicability not only in the U.S. but also across a wide range of habitats sawfish may occupy.

## 2 Methods

### 2.1 In Situ Data Collection

**FIGURE 1:**
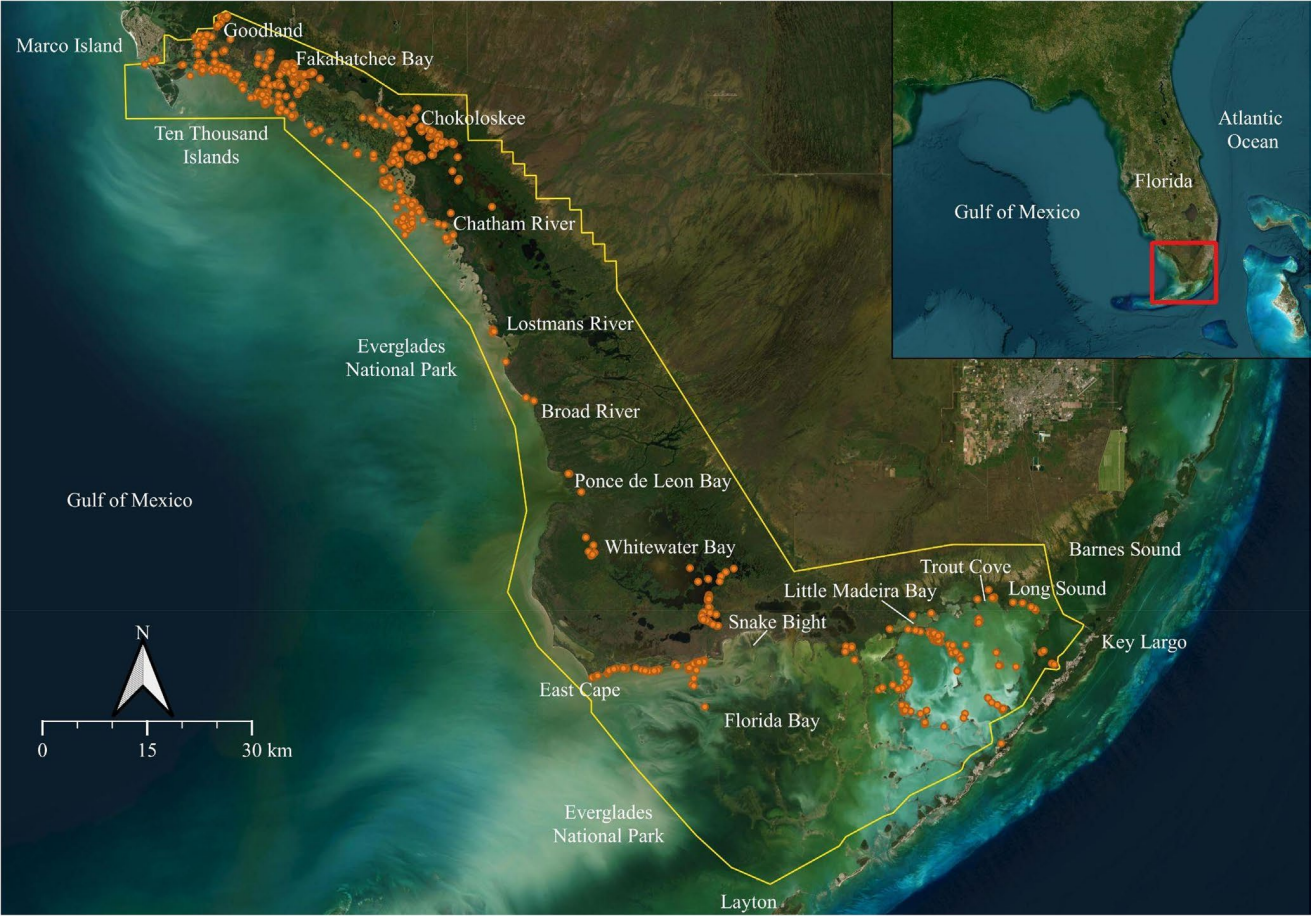
Sampling locations (orange circles) from NOAA Fisheries Service juvenile smalltooth sawfish gillnet survey from 2009 to 2019. Critical Habitat for juvenile smalltooth sawfish is outlined in yellow.

NOAA Fisheries Service‐Panama City Laboratory initiated a scientific gillnet survey in 2009 to monitor the relative abundance and distribution of juvenile smalltooth sawfish (≤ 220 cm stretched total length [STL]) in the Ten Thousand Islands/Everglades Unit (TTI/EU; 2505 km^2^) of sawfish Critical Habitat in southwest Florida (NMFS 2009; Norton et al. 2012; Figure 1). Sampling employed a haphazard design (Andrew and Mapstone 1987), with gillnets set opportunistically in suitable habitats identified during sampling, such as shallow mangrove‐fringed mud flats. Sampling included fixed areas selected based on public encounter records from previous surveys dating back to 1998 where sawfish have been encountered most frequently (Wiley and Simpfendorfer 2010; Simpfendorfer, Wiley, and Yeiser 2010). Although these were fixed areas, they encompassed a wide variety of mangrove habitats, all of which were targeted during each survey. Gillnets used were 1.5 m deep and made of monofilament, with stretch meshes of 7.6 cm or 10.2 cm, and lengths of either 30.5 m or 61.0 m. The nets were primarily set perpendicular to the shore, with soak times ranging from 30 min to 1 h, and checked every 30 min or upon observing a captured sawfish. Captured sawfish were measured, tagged, and released (for detailed survey methods see Kroetz, Carlson, and Grubbs 2023). Data from 2009 to 2019 were included in the analysis, as surveys from 2020 to 2021 were drastically limited due to COVID‐19 restrictions (Kroetz, Carlson, and Grubbs 2023).

Abiotic environmental conditions measured concurrently with gillnet sets were used as explanatory variables in the model (Table 1). Mangrove properties were measured at each gillnet set from 2012 to 2017, where applicable. Counts of red mangrove prop roots and black mangrove ( *Avicennia germinans* ) pneumatophores were taken within a 1.5 m^2^ quadrat, and canopy overhang (cm) was measured from the first mangrove root to the edge of the canopy (Kroetz, Carlson, and Grubbs 2017). In cases where data on mangrove properties were not available, average values from adjacent sampled locations within 1.0 km^2^ area, and the average home range of juvenile smalltooth sawfish (Hollensead et al. 2016) were used.

**TABLE 1:** Explanatory environmental and gear variables that were used in boosted regression tree species distribution models.

Explanatory variable	Description	Source
Daylength*	Analog for season (max daylength = summer)	*daylength* R code (Dedman 2018)
Depth.M*	Average depth of sampling location (m)	In situ; (Becker et al. 2009) (ERDDAP)
DistanceToMangroveKm*	Distance gillnet set was made to the nearest mangrove (km)	Calculated from custom raster
DistanceToShoreKm*	Distance gillnet set was made to shoreline (km)	Calculated from custom raster
DO.MgL	Surface dissolved oxygen (mg/L)	In situ; data sparse
Gear.Soak.Time	Total time gear was soaked in the water (hours)	In situ
Grain.Size.MM.Log*	Log of substrate grain size (mm) of bottom type descriptor (sand, mud, shell, mud mix, etc.)	In situ; (National Ocean Service 2013)
Mesh.Inch	Diameter of gillnet mesh (inch)	In situ
Net.length.ft	Length of gillnet (ft)	In situ
Overhang	Distance of mangrove canopy extending over gillnet (cm)	In situ
Oyster	Oyster substrate present; binary data (0,1)	In situ
Pneumatos	Black mangrove pneumatophores; count within the 1.5 m^2^ quadrat	In situ
Prop. Root	Red mangrove prop roots; count within the 1.5 m^2^ quadrat	In situ
Region	TTINWR, NENP, SENP	In situ
Salinity*	Sea Surface salinity	In situ; (Fore et al. 2016) (JPL CAP SMAP)
Secchi.Depth.M*	Water turbidity (m) measured as Secchi depth	In situ; Global Ocean Color
Seagrass	Seagrass substrate present; binary data (0,1)	In situ
Shell	Shell hash substrate present; binary data (0,1)	In situ
Tidal.State	Tidal condition during gillnet set (ebb, flood)	In situ
Water.Temp.C*	Sea Surface temperature (°C)	In situ; (Chin, Vazquez‐Cuervo, and Armstrong 2017) (GHRSST)
Year	Year in which sampling occurred	In situ
NrstMangAGB*	Nearest aboveground biomass of mangroves via remotely sensed and in situ field measurement data	Simard et al. (2019)
RandomVar	Random variable included via gbm.auto to the exploration models consisting of random values uniformly distributed between 0 and 1 of the same length as the response variable data	In situ

*Note:* Variables with an asterisk (*) were the only variables with enough data to be used in the prediction models.

Abbreviations: NENP = northern Everglades National Park; SENP = Southern Everglades National Park including Florida Bay; TTINWR = Ten Thousand Islands National Wildlife Refuge.

### 2.2 External Data Acquisition and Processing

In situ environmental variables can often provide richer data compared to external sources, such as global satellite‐derived products. However, analyses may be limited to examining the significance and relationships of explanatory variables to a response variable (e.g., sawfish catch and abundance), without the ability to make broader predictions. Hereafter, in situ data models are referred to as *exploration models*. For broader predictions across an area at different times, full spatial coverage of all variables is necessary; these are referred to as *prediction models*. To predict sawfish catch‐per‐unit‐effort (CPUE), we modeled relationships between observed sawfish CPUE and explanatory in situ environmental variables. Our prediction area encompassed the TTI/EU of designated Critical Habitat, extending from Marco Island to Barnes Sound in Florida Bay, and covered broad geographical areas that are conceptually distinct habitats: Ten Thousand Islands National Wildlife Refuge, northern Everglades National Park, and Florida Bay within the southern portion of Everglades National Park (Figure 1). Using ArcMap (ESRI 2012, version 10.1), a mask was created to exclude land and extend approximately 3 km from shore. The temporal extents for the environmental variables were based on typical values at the midpoints of the four seasons: 15 of January (winter), April (spring), July (summer), and October (fall), for the year 2018, following analysis of seasonal average values.

In addition to the in situ variables, the exploration models included a random variable composed of values uniformly distributed between 0 and 1, matched in length to the response variable data. While this approach is not suitable for prediction models, it is a useful technique to assess the value of explanatory variables; any variable performing worse than random should be considered with caution (Soykan et al. 2014).

External environmental data and additional variables were sourced from multiple resources (see Table 1; Appendix S1 for details). Mangrove data, represented as an estimate of satellite‐derived above‐ground biomass (AGB), were obtained from a 30 m resolution, LIDAR‐derived global raster dataset (Simard et al. 2019), though this dataset had many missing values along the shoreline and on land, where they are needed the most. Preliminary analyses indicated that AGB is the best proxy for mangrove variables recorded in situ, although it represents a different aspect of this local flora. Data were cropped to fit the study area and values from the land were appended to the nearest sawfish capture point, or predict‐to‐grid point, ensuring they were no more than 200 m away.

### 2.3 Modeling Approach

To understand the drivers of habitat preference and total predicted space use of smalltooth sawfish in southwest Florida, we used the class‐leading SDM tool, BRTs. They assess the strengths and shapes between explanatory and response variables and predict SDMs based on these learned relationships (Elith et al. 2006). BRTs were used to predict sawfish CPUE and to explore their spatial‐environmental relationships using *gbm.auto* (Dedman et al. 2017) in R (version 4.2.2; R Core Team 2023).

BRTs were fitted using a delta model formulation (Maunder and Punt 2004), owing to the zero‐inflated data (i.e., most samples caught no sawfish). This entails splitting the data into sawfish presence/absence and modeling with a binomial distribution, and separately modeling presence‐only abundances with a Gaussian distribution. The results were then combined into CPUE values (Lo, Jacobson, and Squire 1992). For the exploration models, in‐bag training and testing were conducted using only the observed samples and corresponding explanatory variables to determine the importance and relationships of these variables to the sawfish CPUE response variable (Table 1). To optimize model performance, multiple values for BRT hyperparameters tree complexity (TC), bag fraction (BF), and learning rate (LR) were tested using an iterative approach, and the most performant model runs were identical for exploration and subsequently prediction models: TC: binomial: 6, Gaussian: 9; LR: binomial: 0.01, Gaussian: 0.005; BF: binomial: 0.9, Gaussian: 0.6.

Exploration model performance was then evaluated using metrics that assessed how well the models performed on training data and predictions to the testing data, following 10‐fold cross‐validation. Root mean square error (RMSE) and percentage of deviance explained compared to the null were applied to both binomial (for presence/absence) and Gaussian (for abundance) models. For binomial models, the Area Under the Receiver‐Operator Characteristic curve (AUC; see Parisien and Moritz 2009) and True Skill Score (TSS; Allouche, Tsoar, and Kadmon 2006) were calculated, providing a robust suite of model performance metrics. Once appropriate model performance was achieved for the exploration models, prediction models were conducted on new data beyond the existing range of response data sampling, spatiotemporally extending to the full region and the midpoints of the four seasons noted above, which were available only for the prediction models (Table 1). These models used the explanatory variables that were found by the exploration model to be influential, allowing for performance scores to be compared between the exploration and prediction models, using AUC and TSS.

## 3 Results

### 3.1 Dataset Metrics

The dataset comprised 1718 gillnet sets throughout the TTI/EU over a span of 10 years (Figure 1). Positive catches for smalltooth sawfish occurred in 236 sets (13.7%), totaling 469 sawfish ranging in size from 57.5 to 259.0 cm STL (median 93.5 cm STL). The dataset was dominated by sawfish that were ≤ 150 cm STL (e.g., neonate and young‐of‐the‐year age classes; 89%) and small juveniles comprised 11% of the data. While this study focuses on small juveniles (≤ 220.0 cm STL), five individuals between 222.0 and 259.0 cm STL were also captured during sampling and included in the models. Sawfish were captured in all months with peaks from April to July, and again in October. In the sets where sawfish were caught, 85% of the captures occurred in water temperatures ≥ 24°C, salinities between 3 and 48 , depths between 0.2 and 1.7 m, and dissolved oxygen levels between 0.4 and 9.5 mg/L.

### 3.2 BRT Model Performance

The exploration model, which was built using all available data (Table 1) but could not predict sawfish CPUE to the wider area, had a cross‐validated AUC score of 0.768 and a TSS score of 0.702 for the binomial sub‐model within the delta model, both considered good scores (Lane, Raimondi, and Kudela 2009). The in‐bag Gaussian submodel had a cross‐validated training data correlation score of 0.379. While this cross‐validated score is poor, the delta model approach improved the overall model performance, supporting the inclusion of the Gaussian model. In contrast, the prediction model, built solely using variables available for the broader southwest Florida area, had a cross‐validated AUC score of 0.742 and a TSS score of 0.647 for the binomial submodel within the delta model—considered good and fair scores, respectively (Lane, Raimondi, and Kudela 2009). For the in‐bag Gaussian submodel, the cross‐validated training data correlation score was 0.308.

### 3.3 Exploration Model

#### 3.3.1 Binomial Results

**FIGURE 2:**
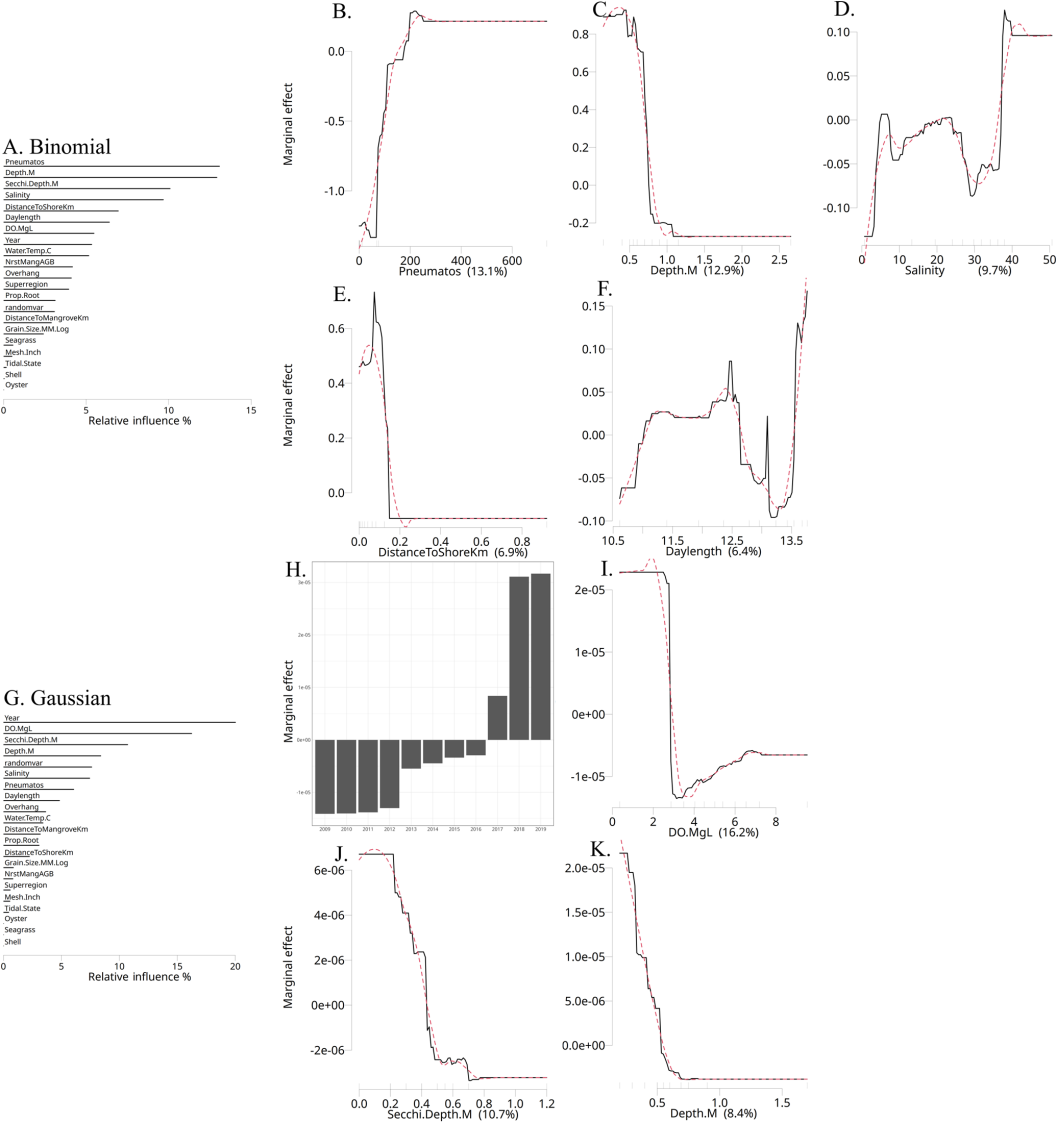
Bar plots and partial dependence plots illustrate the relative influence contributions (%) of explanatory variables for both the exploration boosted regression tree binomial (A) model, as well as for the exploration Gaussian (G) model. The partial dependence plots (PDP) also depict the shapes of these relationships for the binomial (B–F) and Gaussian (H–K) models. Variables listed below “randomvar” in the bar plots perform no better than by random chance. The red dashed line in the PDPs represents a smoother, likely more ecologically realistic relationship that would be expected with more comprehensive data coverage. The values on the *X*‐axis labels of the PDPs correspond to the relative influence values shown in the bar plots.

The binomial exploration model identifies the in situ variables that influence the presence or absence of sawfish and their interrelationships. Many variables outperformed the random variable, suggesting that juvenile sawfish presence is determined by complex, interconnected factors (Figure 2). Among these, black mangrove pneumatophore count was the most influential variable, with sawfish presence notably lower when there were less than 200 pneumatophores per 1.5 m^2^ (Figure 2B). Depth was also highly influential, with sawfish more frequently found in waters shallower than 0.75 m (Figure 2C). Salinity had a generally positive linear relationship to sawfish presence, although with some variability; there was a small peak at low salinity (~6) and a trough at higher salinities (30–40), before showing peak presence values in very saline waters (> 40) (Figure 2D). However, caution is warranted for values above ~38, as they were based on limited data (see distribution of *x*‐axis rug tick deciles). Secchi depth (i.e., turbidity; Appendix S2) and distance to shore (Figure 2E) exhibited a similar steep threshold as depth, with a preference for nearshore shallows where turbidity was lower. These variables are typically correlated in coastal environments, but as mentioned, this is not a disqualifying statistical issue for BRTs. Sawfish presence generally increased as daylength increased (Figure 2F), then decreased between 12.6 and 13.6 h before a peak above 13.6 h. This pattern corresponds to a positive relationship with sawfish presence from early September to early April, and a negative relationship from early April to early September, except for the peak positive values around the summer solstice from late May to mid‐July (Appendix S6). Sawfish presence rose linearly with increasing dissolved oxygen above 7 mg/L, and similarly rose in step with year with positive presence from 2017 and especially 2018 onwards (Appendix S2). Mangrove overhang, study region, and mangrove prop root density all had a modest influence, while bottom substrate variables were unimportant (Appendix S2). Nearest mangrove aboveground biomass was much less influential than mangrove pneumatophores but more than other mangrove variables (Appendix S2).

#### 3.3.2 Gaussian Results

The Gaussian exploration model identifies the in situ variables that influence the number of sawfish present and their interrelationships. In the model, year, dissolved oxygen, Secchi depth, and depth were the dominant variables. While other variables had some influence, their modest model score and relative influence falling below that of the random variable suggest they should only be considered for assessing general trends (Figure 2; Appendix S3). Year (Figure 2H) emerged as the most influential variable, showing higher sawfish abundance in 2017, and especially in 2018 and 2019. Despite sawfish *presence* rising linearly with dissolved oxygen (Figure 2I; Appendix S2), *abundance* was highest at levels below 3 mg/L, after which it declined precipitously. Both Secchi depth (Figure 2J) and depth (Figure 2K) aligned to the binomial results, indicating higher abundance at lower values for each variable.

### 3.4 Prediction Model

#### 3.4.1 Binomial Results

**FIGURE 3:**
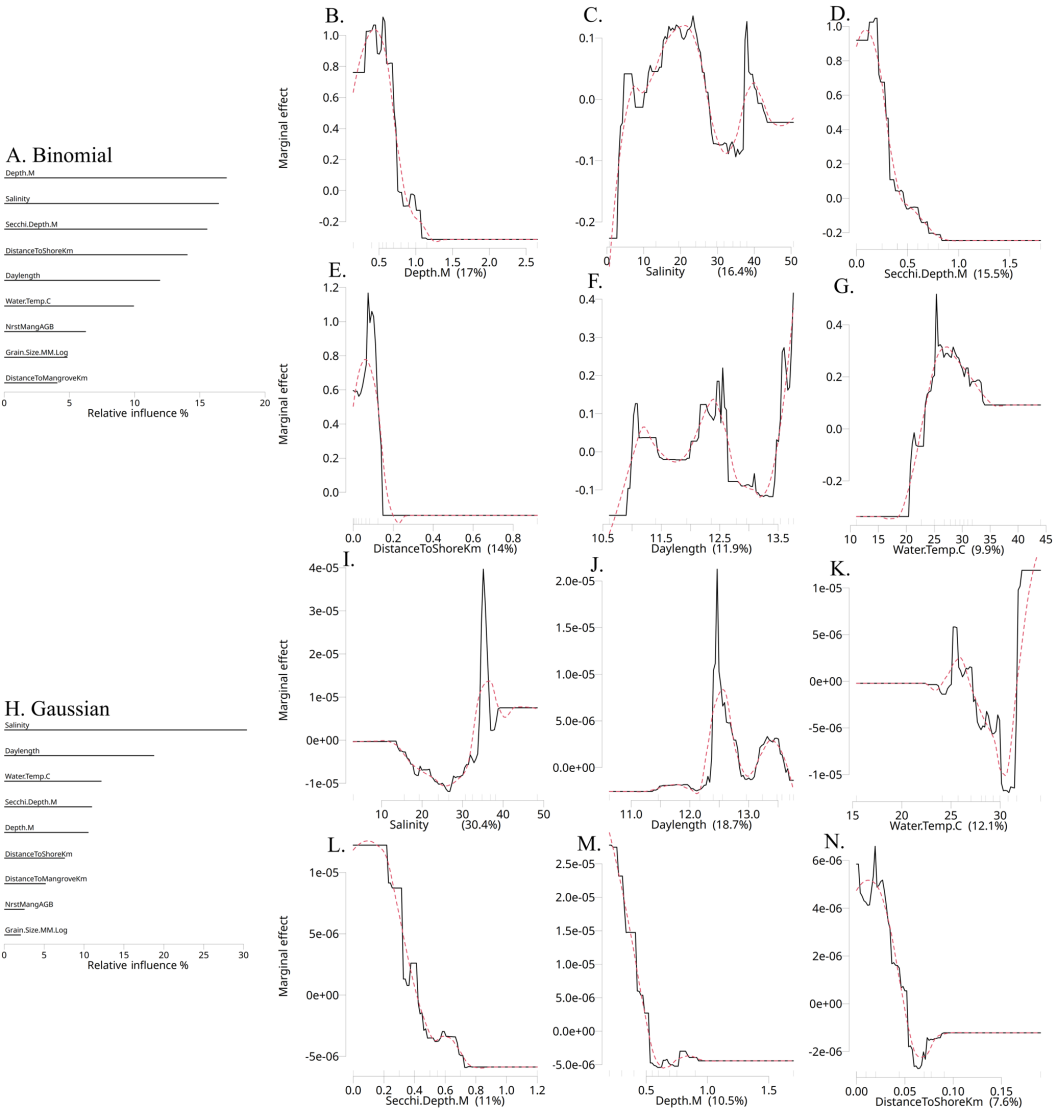
Bar plots and partial dependence plots illustrate the relative influence contributions (%) of explanatory variables for both the prediction‐boosted regression tree binomial (A) model, as well as for the exploration Gaussian (H) model. The partial dependence plots (PDP) also depict the shapes of these relationships for the binomial (B–G) and Gaussian (I–N) models. Variables listed below “randomvar” in the bar plots perform no better than random chance. The red dashed line in the PDPs represents a smoother, likely more ecologically realistic relationship that would be expected with more comprehensive data coverage. The values on the *X*‐axis labels of the PDPs correspond to the relative influence values shown in the bar plots.

**FIGURE 4:**
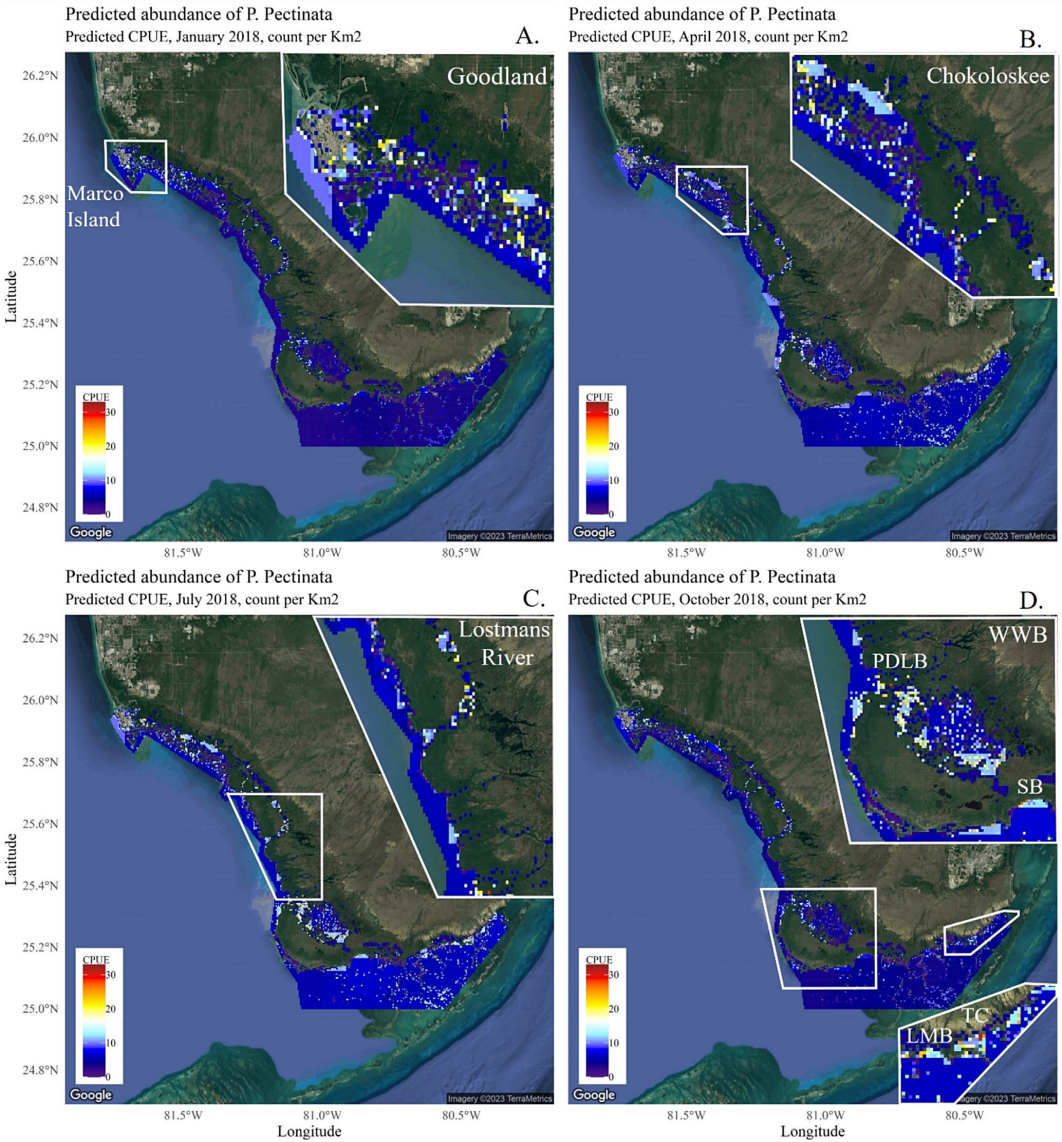
Predictions of smalltooth sawfish CPUE (numbers per hour) across four seasons: January = winter, April = spring, July = summer, and October = fall. Predictions of more smalltooth sawfish presence are denoted by warmer colors of yellow to red and locations with low or zero predictions are denoted by cooler hues of blue. Inset panels show the detail of the July predictions for four key areas: Marco Island/Goodland (A), Chokoloskee area (B), Lostmans River (C), and Whitewater Bay (D) including Ponce de Leon Bay (PLDB), Snake Bight (SB), Little Madeira Bay (LMB), and Trout Cove (TC).

With in situ and random variables removed, the previously influential explanatory variables from the exploration model—such as depth, salinity, Secchi depth, distance to shore, and daylength—retained their significance (Figure 3). The nearest mangrove aboveground biomass increased in relative influence from 4% to 7% (Appendices S2 and S4, respectively), but fell in importance to closer to the bottom. Similar to the exploration model, shallow depth, Secchi depth, and distance to shore continued to correlate to higher sawfish presence (Figure 3B,D,E, respectively). Salinity exhibited a similar dome‐shaped pattern, showing a high marginal effect in the midrange and a decline of around 30–40  (Figure 4C). Daylength (Figure 3F) mirrored the pattern from the exploration model (Appendix S2), while water temperature displayed a dome‐shaped relationship with sawfish presence, peaking at around 27°C (Figure 3G).

#### 3.4.2 Gaussian Results

Salinity emerged as the most influential variable in the Gaussian prediction model with higher abundance observed above 34  (Figure 3I). Daylength exhibited a sharp peak in abundance at 12.5 h, correlating positively to spring (late March to May 1) and fall (mid‐August to October 1) (Figure 3J). These periods aligned to lower presence in the binomial model (Figure 2F). Although there was no sampling in early September, the specific peak in early April may be driven by limited data as the binomial model indicated neutral results during that time (Figure 2F). Water temperature exhibited a similar dome shape around 25°C (Figure 3K), while Secchi depth (Figure 3L), depth (Figure 3M), and distance to shore (Figure 3N) were all consistent with previous results. The importance of the nearest mangrove and distance to the mangrove decreased from the binomial to the Gaussian prediction model (Appendix S5).

#### 3.4.3 Predicted Sawfish Abundance

**FIGURE 5:**
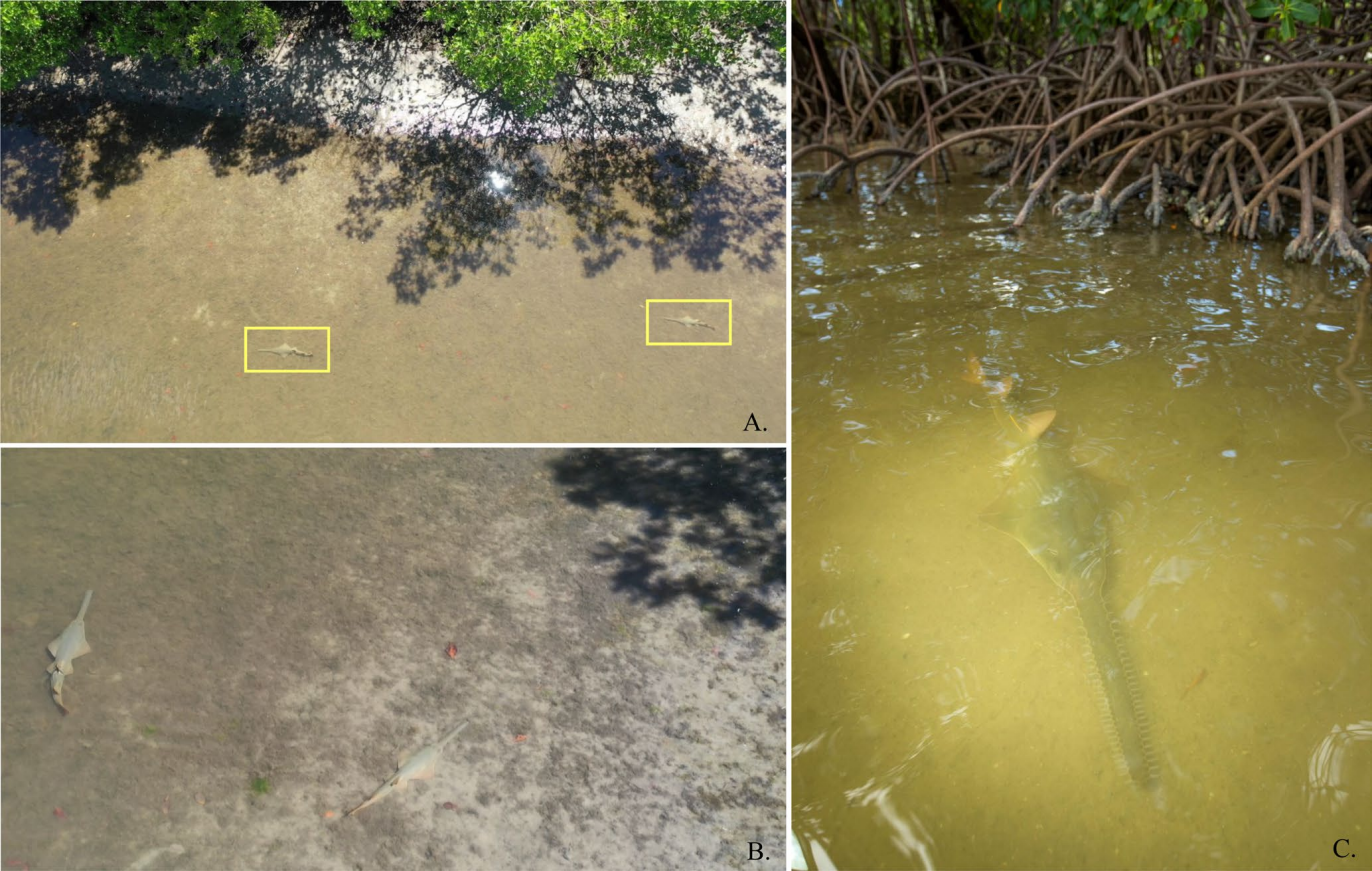
(A, B) Drone footage shows juvenile smalltooth sawfish resting and swimming in shallow (< 0.5 m) waters on a mudflat bordered by red and black mangroves with dense roots. (C) A juvenile resting near the prop roots of red mangroves in its nursery habitat within Everglades National Park. Photo credits: (A) and (B) by Michael Scholl; (C) by Olivier Born under NOAA ESA permit 22078.

Nine explanatory variables with sufficient data were available to predict juvenile smalltooth sawfish abundance throughout the TTI/EU (Table 1). Predictions were made seasonally: January (winter), April (spring), July (summer), and October (fall). The overall highest abundance of juvenile sawfish was predicted for July, followed by April, October, and January (Figure 4). Across all seasons, small pockets of higher abundance were consistent around Marco Island and Goodland (Figure 4A) in the Ten Thousand Islands, around Chokoloskee and Chatham River (Figure 4B), throughout Lostmans River (Figure 4C), Ponce de Leon Bay, Snake Bight, northwest Whitewater Bay, and Long Sound (Figure 4D) in Everglades National Park. Predicted abundance was notably high in these locations as well as in Snake Bight for the month of April, while high predictive abundance in Little Madeira Bay, Trout Cove, Long Sound, and Whitewater Bay occurred in July (Figure 4D). Low predicted abundance occurred for most of the study area, including deeper offshore waters that are not adjacent to mangroves, which typically consist of shallow waters where juvenile sawfish are most commonly found (Figure 5).

### 3.5 Results Synthesis

Overall, sawfish preferred areas of dense black mangrove pneumatophores (> 200 per 1.5 m^2^), depths less than 0.75 m, and saline water (30–40). Sawfish presence and abundance were higher in association with more recent years of the survey. Many explanatory variables retained their importance across the models, although the absence of high‐resolution in situ mangrove data in the prediction models saw other variables take their place. The highest abundance was predicted in a number of inshore bays, coves, and bights, especially in April and July.

## 4 Discussion

Enhancing predictive spatial modeling for rare species is crucial for effective conservation and recovery efforts. Rare species are often data deficient, which, when combined with the lack of high‐resolution environmental data, significantly hinders the predictive performance of spatial models. This study successfully leveraged a long‐term scientific survey along with limited environmental data to understand spatiotemporal habitat use patterns and predict the abundance of a rare and ecologically important species of benthic elasmobranch. While overall predictive performance could be enhanced with higher quality environmental data across broader areas, our models effectively identified locations where juvenile smalltooth sawfish are likely to be found. Given the extensive potential suitable habitat in southern Florida, our findings represent a critical first step towards bolstering essential data for this endangered species by pinpointing key areas of occurrence.

The mangrove ecosystems of Florida, outside the protected TTI/EU Critical Habitat areas, have experienced multiple anthropogenic perturbations resulting in smaller, fragmented forests with altered species compositions (Strong and Bancroft 1994). Identifying these critical habitats is not only vital for the ongoing conservation of smalltooth sawfish but also plays a significant role in broader ecological health. Our study's insight can guide habitat management and restoration efforts, ensuring that conservation strategies are informed by reliable data on where juvenile sawfish thrive. By focusing on areas that support juvenile sawfish, we can prioritize conservation actions, promote the resilience of mangrove ecosystems, and facilitate the recovery of this endangered species. Ultimately, improving spatial modeling and habitat identification contributes to a more comprehensive understanding of the ecological requirements of smalltooth sawfish, fostering better‐informed conservation policies and actions that are crucial for their long‐term survival.

### 4.1 Model Comparisons

Most of the highly influential variables were present in the same order of importance with similar relationship shapes. Year was the most influential variable in the exploration model, and influential in the prediction model. It is reasonable for the year to be a highly influential factor, as the relative abundance of sawfish in the TTI/EU has increased over time, but fluctuated from 2009 to 2019. This is supported by a meta‐analysis of preliminary population trend data of all current smalltooth sawfish abundance data from the U.S. up to 2019 (Carlson et al. 2022), which aligns with the results of this study. The increase in relative abundance can be attributed to successful conservation efforts, notably protection from harvest in Florida in 1992, the 2003 ESA listing of the species (U.S. Federal Register 68 FR 15674), subsequent protective measures, and ongoing conservation initiatives (Brame et al. 2019). Furthermore, designating Ten Thousand Islands and Everglades National Park as critical habitats (Norton et al. 2012) and implementing a ban on gillnets from commercial use in Florida since 1994 (Adams 2000) have likely contributed to this observed increasing trend.

In both models, nearshore shallows with moderate salinities and water temperatures > 24°C were preferred, consistent with existing knowledge about smalltooth sawfish affinity for these environmental conditions (Poulakis et al. 2011; Simpfendorfer et al. 2011; Kroetz, Carlson, and Grubbs 2017). Temperature has emerged as a main driver of habitat use for numerous marine species, a trend that holds true for elasmobranchs and other sawfish species. For example, juvenile largetooth sawfish ( *Pristis pristis* ) have been documented to make small‐scale movements during normal daily or seasonal fluctuations in response to temperature changes (Gleiss et al. 2017; Whitty et al. 2017). The timing of shifts in habitat use noted in these studies may be cued by temperature, demonstrating potential behavioral adaptations to optimize their habitat use. These findings suggest that maintaining suitable thermal regimes and salinity levels in nearshore environments is essential for the conservation of smalltooth sawfish and potentially other elasmobranch species. As climate change continues to impact marine ecosystems, understanding the specific environmental preferences of these species becomes increasingly vital. Moreover, the preference for nearshore shallows highlights the importance of protecting these habitats from anthropogenic pressures, such as coastal development and pollution, which could alter the salinity and temperature profiles critical for the survival and recruitment of sawfish populations.

The exploration model showed sawfish preference for high mangrove density with all mangrove variables contributing. While spatial data on the presence of mangroves were available, data for mangrove density and pneumatophore density were limited, which prevented their inclusion in the exploration model. The absence of mangrove pneumatophores from the prediction model did not result in the replacement of its variability from another source like nearest‐mangrove above‐ground biomass, which was acquired and processed as a lower‐resolution proxy for pneumatophores. Rather, the other oceanographic and geophysical variables became more influential as the absence of the pneumatophore variable only dropped the CV AUC score of the model by −0.026. The correlations between higher predicted sawfish occurrence and factors such as proximity to shore, depth, and mangrove presence support previous research that juvenile smalltooth sawfish rely heavily on mangrove habitat for their nurseries and that these environmental variables are crucial habitat features (Wiley and Simpfendorfer 2007; Simpfendorfer, Wiley, and Yeiser 2010; Poulakis et al. 2011; Simpfendorfer et al. 2011). The negligible effect of the removal of this variable on model performance may be attributed to fragmented mangrove data as spatial coverage was decent but shoreline coverage was lacking. This has likely contributed to the distance to the mangrove variable having a generally low influence in the models. Given the importance of mangroves to sawfish (Norton et al. 2012; Brame et al. 2019; Poulakis and Grubbs 2019), as evidenced in early exploration model iterations that indicated mangrove variables were the most influential on sawfish presence, we suspect this result is due to imperfect data.

Earlier model runs featured a notable decrease in performance from exploration models with all in situ mangrove and gear variables, to exploration models with all in situ mangrove variables, to prediction models with only externally available variables. However, the final production runs no longer experienced this issue; performance scores dipped negligibly from the full in situ variable set to the subset of external variables resulting in good performance scores for both models. While many variables were strongly correlated in some regions, this was not universally true across the study area, where, for example, expansive shallow flats could result in a high distance from shore and increasingly oceanic salinity but a shallow depth. BRTs are robust to autocorrelated variables (Elith, Leathwick, and Hastie 2008; Abeare 2009), and their inclusion affords the models the scope to tease out nuanced independent explanatory‐response variable relationships.

Discrepancies between the predicted abundance maps across seasons were subtle, as the predict‐to areas were largely suboptimal. That is, the poorest representative coverage of explanatory variable data was around inshore, mangrove‐adjacent extreme shallows that juvenile sawfish prefer, and the vast majority of the data were in deeper offshore waters. This is inevitable because nearshore shallows with low Secchi depth are relatively uncommon compared to the vast areas that are further offshore and deeper. This is unlikely to represent a statistical concern that would undermine any conclusions drawn from these results.

The highest predicted CPUE was in July, followed by April, October, and finally January, likely aligning to ambient water temperatures in those months and/or parturition. The increased predicted CPUE of sawfish during these months corresponds with known patterns of parturition. In the Charlotte Harbor Estuary Unit (CHEU), a designated Critical Habitat and nursery area for sawfish in the U.S., parturition occurs from November to July, peaking in April and May (Poulakis et al. 2011). Similarly in the TTI/EU, neonates in this study that showed signs of recent pupping, such as rostral sheaths or open yolk‐sac scars, were typically captured from February to July, while captures of individuals near birth size (≤ 74 cm STL; A. Kroetz unpublished data) peaked in April and May. Sawfish in this study were primarily ≤ 150 cm STL (87%) and were present year‐round, again peaking in April and extending to July, coinciding with higher predicted abundance. Female sawfish are regionally philopatric for parturition and have been found to return to consistent areas within TTI/EU to pup (Smith et al. 2021), and thus likely contributed to higher predicted CPUE. Small pockets of higher predicted CPUE were often consistent around Marco Island and Goodland in the Ten Thousand Islands, as well as in Lostmans River, Snake Bight, northwest Whitewater Bay, and Long Sound in Everglades National Park (see Figures 1 and 4). Lostmans River and Snake Bight have been two areas of higher reported encounters (U.S. Sawfish Recovery Database) for several years but are logistically challenging locations to sample. Whitewater Bay, particularly near Ponce de Leon Bay, is an area of higher predicted abundance that is also difficult to access with current sampling equipment and protocols. All three areas are recognized as important and plans to sample these locations in the future are in place (Kroetz, Carlson, and Grubbs 2023).

Biases in our survey design could have influenced overall inferences from the models. For example, the TTI/EU is a large area (2505 km^2^) of complex, potentially suitable habitat for sawfish, much of which is difficult to access by either boat or land. The survey used in this study was created to monitor the relative abundance and distribution of the juvenile smalltooth sawfish population throughout the TTI/EU, and sampling in areas where sawfish have been most frequently captured was a priority. Fixed and opportunistic sampling was also influenced by the accessibility of potential habitat, which was poor in many areas (e.g., Lostmans and Broad rivers). Repeatedly sampling these locations where sawfish were often captured at each sampling event, compounded with a very large overall sampling area that was difficult to comprehensively cover with the funds available, could have inflated CPUE estimates in these locations at the expense of the less‐sampled locations, biasing the model. However, many of these fixed areas represent a diverse mangrove canopy and sampling was distributed throughout these areas during each sampling event. We acknowledge that these caveats represent some threat to the overall integrity of the results; however, we conclude that our long‐term dataset on a rare species is invaluable and has provided pivotal information on suitable habitats for the species.

### 4.2 Study Challenges

Our study faced challenges and limitations that precluded high‐resolution modeling of the presence and abundance of a rare and endangered species. The primary limitation in our modeling was data availability and acquisition for key environmental predictors at high resolutions across the study area, particularly mangrove‐related variables. Our scientific survey data demonstrates that juvenile smalltooth sawfish are primarily encountered in very shallow, warm waters on mangrove shorelines, typically less than 30 m from the shore (Kroetz, Carlson, and Grubbs 2023). Thus, corresponding environmental data outside of our sampling locations needed to be in this high resolution to reliably predict sawfish presence. However, many of the variables were missing for large portions of the prediction study area and within 30 m or less of shoreline, precluding fine‐scale predictions of sawfish presence. Despite good overall performance by the prediction model, predictions are likely to be powered by fewer data in certain inshore areas, and thus less robust in those places. As a rare and protected species, sawfish is a data‐limited species by nature, exacerbated by the absence/low spatiotemporal resolution and quality of their explanatory environmental variables. This likely culminated in suboptimal predictions throughout the study area, which is an important area to model as it is a nursery and Critical Habitat for juvenile smalltooth sawfish (Norton et al. 2012). Though predictions are not fine‐scale, the results presented here are of vital importance to the continued conservation efforts and habitat protections for this species.

Data availability and acquisition is a long‐standing (Iverson 2007) and ongoing (Michener 2015) problem across many fields in ecology, ultimately delaying conservation, to the detriment of rare species. The data acquisition portion of this study was extremely lengthy, despite Florida being a global hotspot of elasmobranch research, hosting multiple well‐funded university, state, and federal institutions with well‐connected overarching data surveying and research goals, and vast conservation‐designated areas. Rare species in remote areas with little to no survey and external environmental data will be decidedly harder to model. Over a third of elasmobranchs are threatened (Dulvy et al. 2021), increasingly addressed via spatial conservation (e.g., Davidson and Dulvy 2017), requiring analyses of essential habitat (e.g., Important Shark and Ray Areas [ISRA] project, Hyde et al. 2022), requiring ample data. Data availability and ease of processing can thus prevent the successful designation of essential habitats needed for the conservation of a species.

Mangroves are among the most diverse and threatened ecological systems globally (Alongi 2002; Valiela, Bowen, and York 2001; Duke et al. 2007; Dabalà et al. 2023), providing foundation habitats for most species of sawfish (Poulakis and Grubbs 2019), and are important habitat for many species of elasmobranchs (White and Potter 2004; Kanno et al. 2023). Despite their importance, global coverage of high‐resolution data for all species of mangroves has been difficult to acquire. Insufficient high‐resolution mangrove data creates many problems in conservation as many rare and threatened species depend on mangroves at some point in their lifetime. For example, four species of critically endangered sawfish depend on mangrove habitats across the globe as nurseries (Whitty et al. 2008; Morgan et al. 2011; Norton et al. 2012; Elhassan 2018). Similarly, many global species of sharks and rays on the IUCN Red List depend on mangroves during some stage in their life history (Cerutti‐Pereyra et al. 2014; Oh et al. 2017; Llerena et al. 2015), as well as teleosts such as the Goliath grouper ( *Epinephelus itajara* ), which shares mangrove nursery habitat within our study area (Koenig et al. 2007). Mangrove data were available at a 30 m resolution though highly fragmented and missing data at the shoreline, and even for pixels fully on land, creating insufficiencies within our models. The exploration model supports previous research that mangroves are crucial for juvenile sawfish; however, predictions based on the available datasets for potential habitats performed less effectively than they could have, given the data quality. While we cannot compare the performance scores of the current prediction model against one powered by high‐resolution, full‐coverage mangrove data (we did not have access to such data), the limitations of the available datasets inevitably compromise the precision of fine‐scale inshore predictions. This suggests that, while our broader conclusions are likely valid, further in situ ground‐truthing is recommended to verify high CPUE locations before implementing further conservation policies, such as preventing recreational fishing within certain areas of ENP. Additionally, intentionally sampling transition areas with moderate predicted CPUE, especially in more challenging locations, and incorporating in situ environmental sampling, may enhance the models' ability to distinguish between favorable and unfavorable habitats, resulting in a more comprehensive understanding of these habitats.

There have been recent developments in mangrove data, with a global dataset of mangrove extent and change collected from L‐band Synthetic Aperture Radar and optical satellite data (Bunting et al. 2018), global 30 m resolution LiDAR (Simard et al. 2019), a Google Earth Engine tool (Yancho et al. 2020), and various similar image processing advancements (Giri 2021). However, these datasets did not provide sufficient resolution for our study area, and many are highly technically complex to use, often requiring support from product owners, which may not be available. We are optimistic that advances in this field will improve both the accessibility and processing of these data. Remote sensing data are essential tools for SDMs in conservation efforts (McCauley et al. 2024), but inadequate resolution and coverage can impede modeling and management outcomes, especially when key habitats for the species of interest are located in small areas, at land/water interfaces, or both. Therefore, improving the availability and usability of high‐resolution remote sensing data is crucial for better management of vulnerable marine species inhabiting restricted inshore environments.

### 4.3 Conservation Implications

Predictive spatial modeling is a vital tool for the conservation of rare species, and this study underscores the crucial need for high‐quality environmental data to make accurate predictions about their distribution at both fine and broad scales. Rare species, such as the smalltooth sawfish, often have specialized habitat preferences (Spitale 2012), making it essential to protect the habitats where these species thrive to ensure successful conservation efforts and maintain ecosystem biodiversity (Lawler et al. 2003). Countries with limited data and sampling resources face greater challenges in conserving these vulnerable populations. Therefore, we advocate for data sharing among researchers, facilitating the easy acquisition of environmental and oceanographic data that can benefit multiple studies and bolster conservation initiatives.

This study particularly emphasizes the significance of shallow, mangrove‐fringed inshore areas for juvenile smalltooth sawfish (NMFS 2009; Norton et al. 2012; Wiley and Simpfendorfer 2010; Poulakis et al. 2011; Simpfendorfer et al. 2011; Kroetz, Carlson, and Grubbs 2017) and supports evidence of the species' gradual recovery in the TTI/EU (Carlson et al. 2022) following protective measures. These findings highlight the need to continue to conserve these critical habitats, especially in light of their proximity to anthropogenic influences. Preventing mangrove loss due to human development is essential across the range of smalltooth sawfish, particularly in vulnerable areas like the Charlotte Harbor Estuary and the nearby Bahamas. By identifying and prioritizing these key habitats for conservation, we can take actionable steps to protect the smalltooth sawfish and enhance the resilience of the ecosystems they inhabit. Ensuring the availability of high‐quality data is fundamental not only for the effective management of this endangered species but also for the broader goal of preserving marine biodiversity.

## Author Contributions

**Andrea M. Kroetz:** conceptualization (supporting), data curation (lead), formal analysis (equal), investigation (lead), methodology (equal), project administration (lead), resources (lead), validation (equal), visualization (equal), writing – original draft (lead), writing – review and editing (equal). **Simon Dedman:** data curation (supporting), formal analysis (lead), methodology (equal), validation (equal), visualization (equal), writing – original draft (supporting), writing – review and editing (equal). **John K. Carlson:** conceptualization (lead), funding acquisition (lead), writing – review and editing (supporting).

## Conflicts of Interest

The authors declare no conflicts of interest.

## Supporting information


Appendix S1.
**Appendix S2**.
**Appendix S3**.
**Appendix S4**.
**Appendix S5**.
**Appendix S6**.
**Appendix S7**.

## Data Availability

BRT modeling procedures reported followed Dedman et al. (2017) (https://doi.org/10.1371/journal.pone.0188955) and custom scripts are hosted at Zenodo: https://zenodo.org/doi/10.5281/zenodo.1275290. Due to the smalltooth sawfish being endangered and the sensitive nature of the data, we did not include geolocation data (presence records) of where the sawfish were found. External environmental parameters used in the model are publicly available as listed in Table 1 and in references and their processing is described in the “sawfish_processing_code” R script in the Zenodo repository.
