# Supplementary material for: Predictive Modeling of Juvenile Smalltooth Sawfish Habitats: Challenges and Opportunities for Conservation
Source: Ecol Evol. 2025 Jan 21;15(1):e70592. doi: 10.1002/ece3.70592 (PMC11747141; doi:10.1002/ece3.70592)
Supplement: Supplementary file 1 — Appendix S1. Appendix S2. Appendix S3. Appendix S4. Appendix S5. Appendix S6. Appendix S7. [file ECE3-15-e70592-s001.docx]

**Supplementary Material**

**Predictive modeling of juvenile smalltooth sawfish habitats: challenges and opportunities for conservation**

**Appendix S1. Details on data acquisition of environmental variables & reasoning for inclusion in the models.**

Prediction of sawfish abundance to spatial areas required defining the area sampled per gillnet set and was derived from several metrics. Maximum swimming speed of juvenile smalltooth sawfish (≤175cm stretched total length [STL] per Hollensead et al., 2016) has been documented at 6.1 m/min (Hollensead et al., 2016), thus approaching from either side of the gillnet, the along-coastline area sampled per set was calculated as swimming speed multiplied by gillnet soak time. Sawfish counts recorded per set were then divided by this result to return the sawfish catch-per-unit-effort (CPUE) per m2.

Depth data were acquired from the ERDDAP Coastwatch dataset (Tozer et al., 2019) and clipped to our spatial extent mask. This served as our highest resolution gridded basemap (i.e., 15 arc seconds) to which data from other explanatory variables were appended. Water temperature data were acquired from the Global Foundation Sea Surface Temperature Analysis (v4.1) dataset (JPL MUR MEaSUREs Project. 2015; Chin et al. 2017), converted from point grids to Voronoi polygons (squares) in QGis v3.30.2 (QGIS Development Team, 2023) and appended to the depth grids. Salinity data were acquired from the JPL SMAP Level 2B CAP Sea Surface Salinity V4.2 Validated Dataset (JPL Climate Oceans and Solid Earth group. 2019; Fore et al. 2016), again converted to Voronois and appended to the depth grids. Turbidity (Secchi Transparency Depth) data were acquired from Copernicus-GlobColour Bio-Geo-Chemical daily dataset (Global Ocean Color), with values extracted for the 4 projected seasonal dates and appended to depth grids based on proximity, using a custom script in R. Dissolved oxygen data that were found were too sparse to be usable.

Year was included as a variable (2009-2019) as it can index broadscale environmental patterns missed by other variables, e.g., climate change and macroscale events like the El Nino Southern Oscillation (ENSO). Lack of a clear trend (for climate change) or discrete peak/trough (ENSO) will give year a low influence, after which it may be removed. Seabed descriptions from NOAA/NOS/USCGS (National Ocean Service 2013) were converted to substrate grain size range midpoints, using MNCR/Wentworth classification (e.g., Valentine et al., 2019), then naturally logged in R. Since the input data are points covering a limited extent of the survey area, these were converted to Voronoi polygons each with a maximum extent approximating the maximum spacing between successive points to avoid extrapolating values over unrepresentative large extents. Then, those values were appended to survey sampling points overlapping the Voronoi polygons. Daylength was calculated using the *daylength* package in R (Dedman, 2018), using *lutz* (Teucher, 2023) and *suncalc* (Thieurmel & Elmarhraoui, 2022) to calculate sunlit day duration from geoposition and date. Distance to shore was calculated against a high-resolution coastline raster using the *gridDistance* function from the *raster* package in R (Hijmans, 2023). Distance to mangrove was calculated similarly, against a mangrove raster generated from data acquired from the Florida Fish and Wildlife Conservation Commission’s Mangrove Habitat in Florida database (FWC, 2015). Depths and distances to shore/mangroves were unchanged seasonally.

**Appendix S2. Binomial exploration model Partial Dependence Plots (PDP) of remaining variables not included in the main manuscript.**


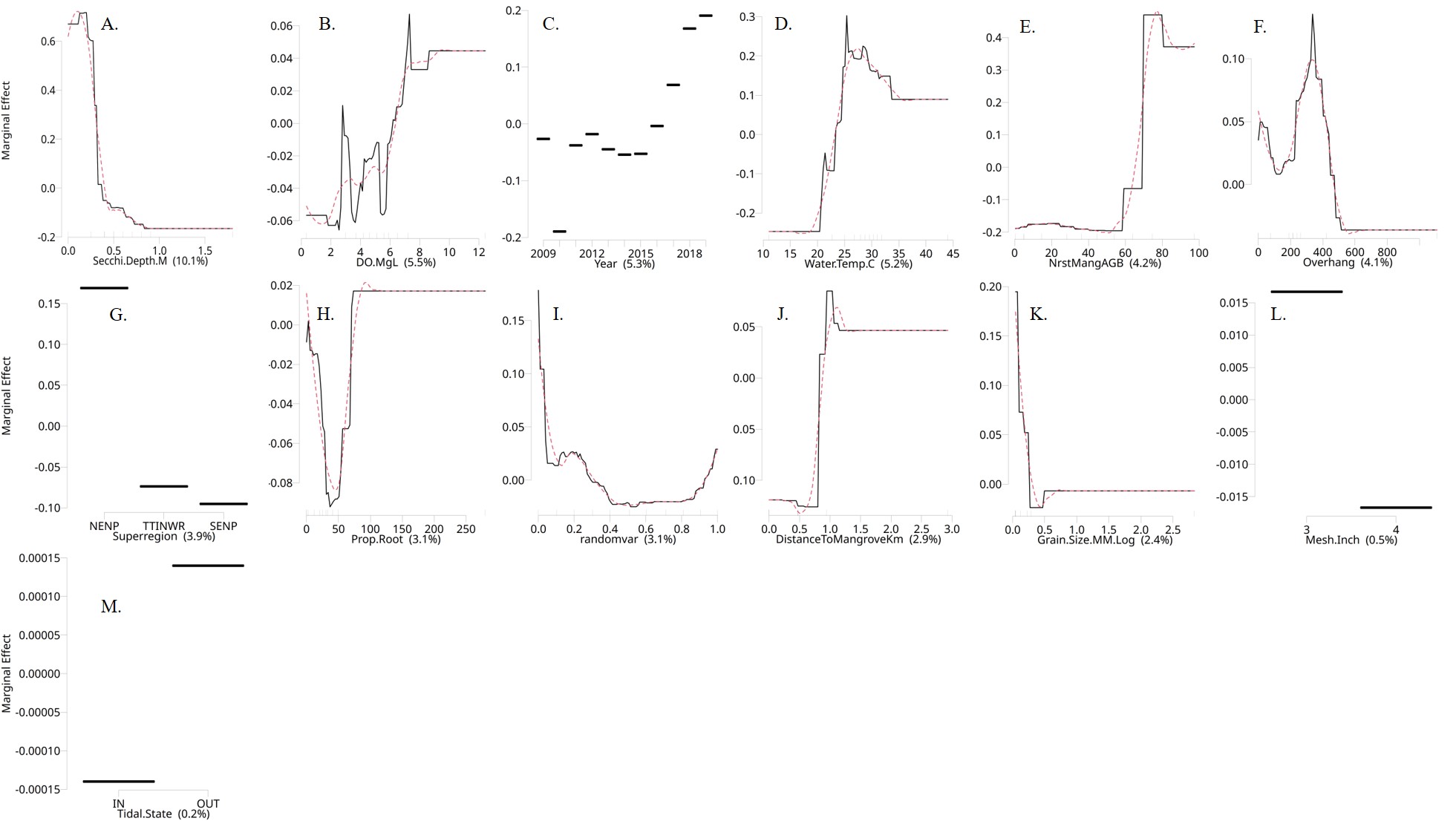


Partial dependence plots (PDPs) illustrate the relative influence contributions (%) of explanatory variables for the binomial exploration boosted regression tree model. The PDPs depict the shapes of these relationships and are sown in decreasing order of relative importance. The red dashed line in the PDPs represents a smoother, likely a more ecologically realistic relationship that would be expected with more comprehensive data coverage. The values on the X-axis labels of the PDPs correspond to the relative influence values shown in the bar plots.

**Appendix S3. Gaussian exploration model PDPs of remaining variables not included in the main manuscript.**
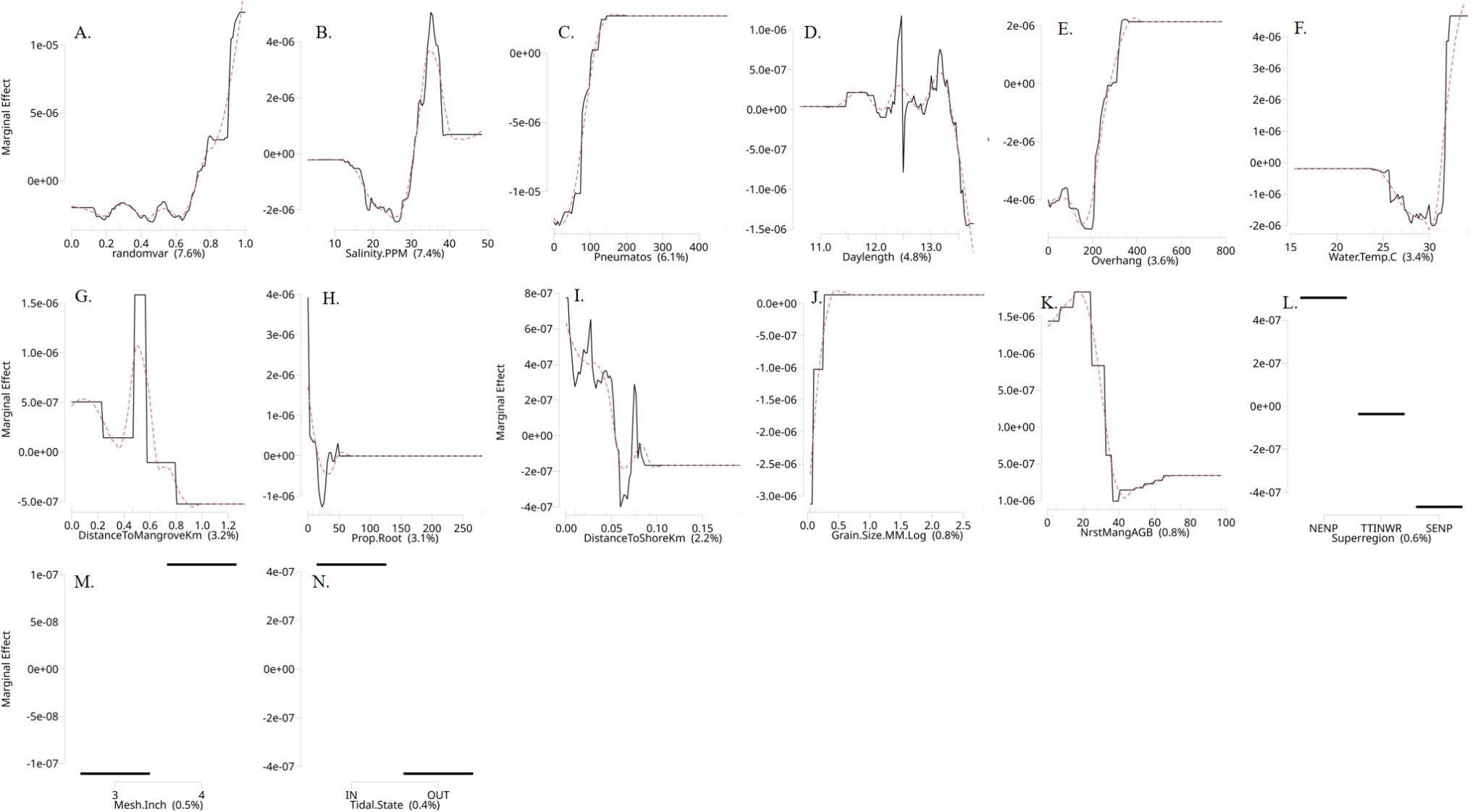


Partial dependence plots (PDPs) illustrate the relative influence contributions (%) of explanatory variables for the Gaussian exploration boosted regression tree model. The PDPs depict the shapes of these relationships and are shown in decreasing order of relative importance. The red dashed line in the PDPs represents a smoother, likely a more ecologically realistic relationship that would be expected with more comprehensive data coverage. The values on the X-axis labels of the PDPs correspond to the relative influence values shown in the bar plots.

**Appendix S4. Binomial prediction model PDPs of remaining variables not included in the main manuscript.**


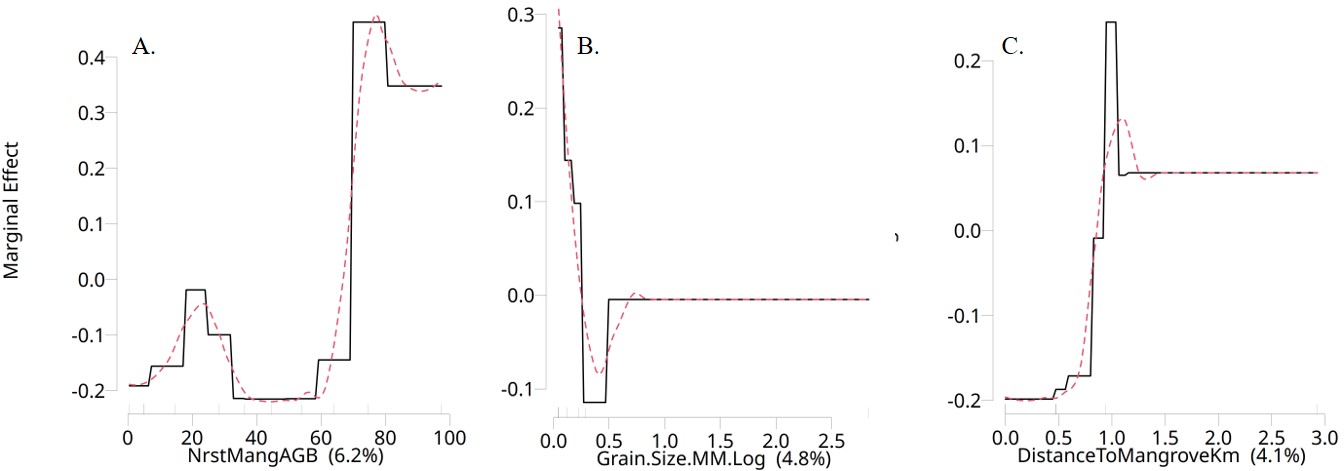


Partial dependence plots (PDPs) illustrate the relative influence contributions (%) of explanatory variables for the binomial prediction boosted regression tree model. The PDPs depict the shapes of these relationships and are shown in decreasing order of relative influence. The red dashed line in the PDPs represents a smoother, likely a more ecologically realistic relationship that would be expected with more comprehensive data coverage. The values on the X-axis labels of the PDPs correspond to the relative influence values shown in the bar plots.

**Appendix S5. Gaussian prediction model PDPs of remaining variables not included in the main manuscript.**


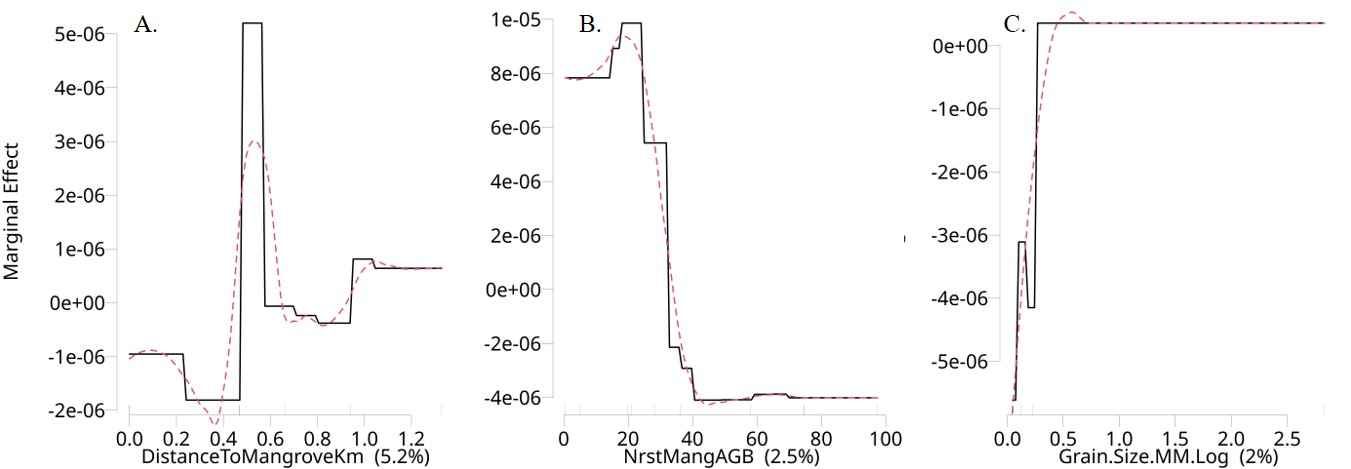


Partial dependence plots (PDPs) illustrate the relative influence contributions (%) of explanatory variables for the Gaussian prediction boosted regression tree model. The PDPs depict the shapes of these relationships and are shown in decreasing order of relative influence. The red dashed line in the PDPs represents a smoother, likely a more ecologically realistic relationship that would be expected with more comprehensive data coverage. The values on the X-axis labels of the PDPs correspond to the relative influence values shown in the bar plots.

**Appendix S6. Sawfish presence as indicated by daylength (i.e., proxy for season)**


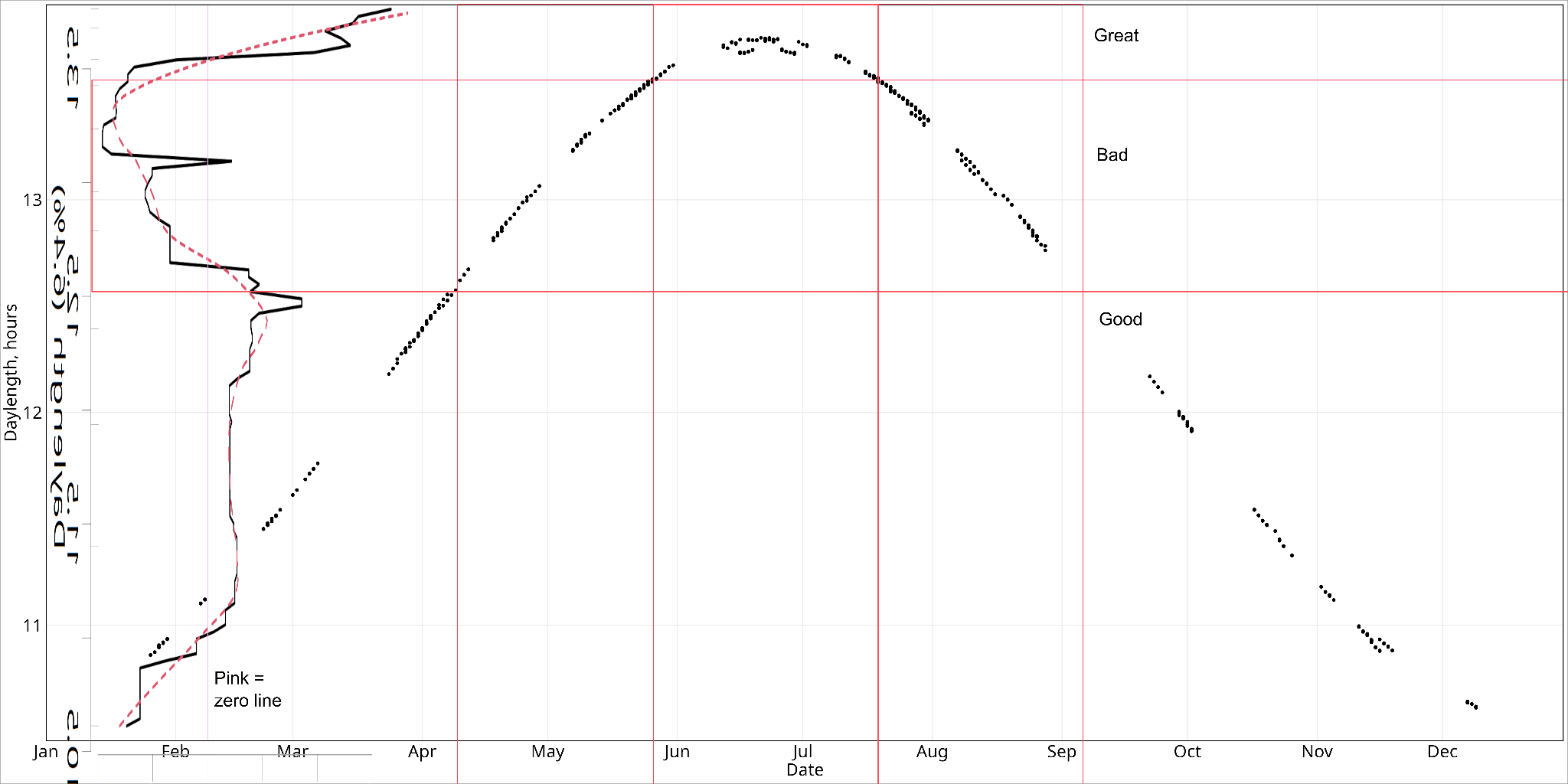


Daylength hours (y) against yearday (x), with daylength partial dependence plot (PDP) values flipped and inverted and superimposed onto the Y-axis. Regions of high marginal effect from the PDP are marked as red sections on the Y-axis, then marked where they intersect with the daylength/date curve and labeled as regions, showing late-May to mid-July is associated with high marginal effect, as is early-September to early-April. Whereas early-April to late-June, and mid-July to early-September are associated with low marginal effect.

**Appendix S7. Unrepresentativeness**
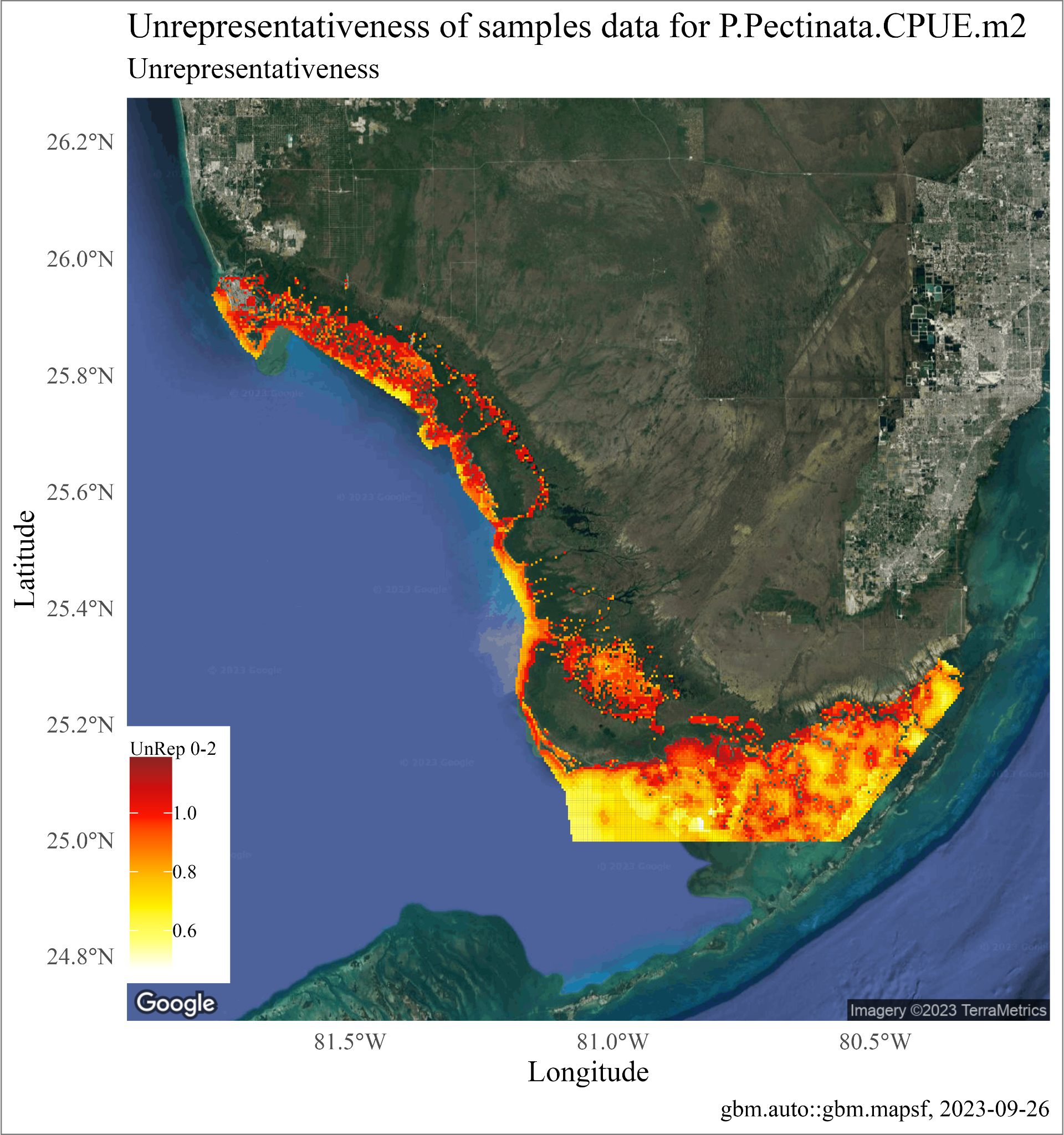


Unrepresentativeness measures how effectively the in situ/sample data reflect the complete range of values found in the ex-situ/grid data. The UnRep score is calculated as the sum of the individual UnRep scores for all variables. Poor scores for coastal and inland waterways are likely due to inadequate ex situ coverage of dissolved oxygen and salinity, for which high-resolution nearshore data were sparse. Additionally, the sampling predominantly occurred very close to shore, resulting in a dataset heavily influenced by shallow, nearshore conditions with low Secchi depth and proximity to mangroves, which represent only a subset of the broader range of values throughout the entire study area.

**References**

Chin, T.M, Vazquez-Cuervo, J., & Armstrong, E.M. (2017). A multi-scale high-resolution analysis of global sea surface temperature*. Remote Sensing of Environment, 200*, pp. 154-169. <https://doi.org/10.1016/j.rse.2017.07.029>

Dedman, S. (2018). Daylength: Calculate day length from date, lat & lon values. <https://github.com/SimonDedman/daylength>

Florida Fish and Wildlife Conservation Commission. (2015). Mangroves Florida. Online database: <https://geodata.myfwc.com/datasets/myfwc::mangrove-habitat-in-florida/explore>.

Fore, A.G., Yueh, S.H., Tang, W., Stiles, B.W. & Hayashi, A.K. (2016). Combined active/passive retrievals of ocean vector wind and sea surface salinity with SMAP. IEEE *Transactions on Geoscience and Remote Sensing, 54(12)*, pp.7396–7404. DOI:10.1109/TGRS.2016.2601486

Global Ocean Colour (Copernicus-GlobColour), Bio-Geo-Chemical, L3 (daily) from Satellite Observations (1997-ongoing). Copernicus Marine Service Information (CMEMS). Marine Data Store (MDS). <https://doi.org/10.48670/moi-00280> (Accessed on 2023-05-23)

Hijmans, R. (2023). Raster: Geographic Data Analysis and Modeling. R package version 3.6-26, [https://CRAN.R-project.org/package=raster](https://cran.r-project.org/package=raster).

Hollensead, L.D., Grubbs, R.D., Carlson, J.K., & Bethea, D.M. (2016). Analysis of fine-scale daily movement patterns of juvenile *Pristis pectinata* within a nursery habitat. *Aquatic Conservation: Marine and Freshwater Ecosystems 26*, 492–505.

JPL MUR MEaSUREs Project. (2015). GHRSST Level 4 MUR Global Foundation Sea Surface Temperature Analysis. Ver. 4.1. PO.DAAC, CA, USA. Dataset accessed [2019-12-14] at <https://doi.org/10.5067/GHGMR-4FJ04>

JPL Climate Oceans and Solid Earth group. (2019). JPL CAP SMAP Sea Surface Salinity Products. Ver. 4.2. PO.DAAC, CA, USA. Dataset accessed [2019-12-14] at <https://doi.org/10.5067/SMP42-2TOCS>

National Ocean Service (2013): NOAA/NOS and USCGS Seabed Descriptions from Hydrographic Surveys. National Geophysical Data Center, NOAA, DOI:10.7289/V5BG2KWG [Accessed on 2021-09-16]

QGIS Development Team (2023). QGIS Geographic Information System. Open Source Geospatial Foundation Project.<http://qgis.org>

Teucher, A. (2023). *lutz*: Look Up Time Zones of Point Coordinates. R package version 0.3.2, [https://CRAN.R-project.org/package=lutz](https://cran.r-project.org/package=lutz).

Thieurmel, B., & Elmarhraoui, A. (2022). *suncalc*: Compute Sun Position, Sunlight Phases, Moon Position and Lunar Phase. R package version 0.5.1, [https://CRAN.R-project.org/package=suncalc](https://cran.r-project.org/package=suncalc).

Tozer, B., Sandwell, D., Smith, W.H.F., Olson, C., Beale, J.R., & Wessel, P. (2019). Global bathymetry and topography at 15 arc sec: SRTM15+. *Earth and Space Science, 6*, 1847–1864. <https://doi.org/10.1029/2019EA000658>

Valentine, P.C. (2019). Sediment classification and the characterization, identification, and mapping of geologic substrates for the glaciated Gulf of Maine seabed and other terrains, providing a physical framework for ecological research and seabed management. *U.S. Geological Survey Scientific Investigations Report (No. 2019*–*5073*). <https://doi.org/10.3133/sir20195073>
